# Down-regulation of long non-coding RNA SNHG14 protects against acute lung injury induced by lipopolysaccharide through microRNA-34c-3p-dependent inhibition of WISP1

**DOI:** 10.1186/s12931-019-1207-7

**Published:** 2019-10-28

**Authors:** Jinyuan Zhu, Jijia Bai, Shaojin Wang, Hui Dong

**Affiliations:** 1 Department of Critical Care Medicine, General Hospital of Ningxia Medical University, Yinchuan, 750004 People’s Republic of China; 2 Department of Respiratory and Critical Care Medicine, General Hospital of Ningxia Medical University, Yinchuan, 750004 People’s Republic of China; 3 Center of Research Equipment Management, General Hospital of Ningxia Medical University, No. 804, Shengli South Street, Xingqing District, Yinchuan, 750004 Ningxia Hui Autonomous Region People’s Republic of China

**Keywords:** Long non-coding RNA SNHG14, MicroRNA-34c-3p, Wnt1 inducible signaling pathway protein 1, Acute lung injury, Lipopolysaccharide

## Abstract

**Background:**

Accumulating evidence has shown the important roles of long non-coding RNAs (lncRNAs) in acute lung injury (ALI). This study aimed to investigate the potential role of lncRNA small nucleolar RNA host gene 14 (SNHG14) in lipopolysaccharides (LPS)-induced ALI.

**Methods:**

Expression of SNHG14, microRNA-34c-3p (miR-34c-3p) and Wnt1 inducible signaling pathway protein 1 (WISP1) in LPS-exposed mouse alveolar macrophages (MH-S) and lung tissues from mice with LPS-induced ALI was determined by reverse transcription quantitative polymerase chain reaction. The interactions among SNHG14, miR-34c-3p and WISP1 were analyzed by dual-luciferase reporter and RIP assays. Using gain-of-function or loss-of-function approaches, the contents of proinflammatory proteins were determined and MH-S cell viability was assessed to evaluate the in vitro functions of SNHG14, miR-34c-3p and WISP1, and wet/dry weight ratio and proinflammatory proteins in lung tissues were determined to assess their in vivo effects.

**Results:**

SNHG14 and WISP1 expression was increased, while miR-34c-3p was decreased in ALI models. SNHG14 bound to miR-34c-3p, resulting in impaired miR-34c-3p-dependent down-regulation of WISP1. Both SNHG14 silencing and miR-34c-3p over-expression reduced the levels of proinflammatory proteins IL-18, IL-1β, TNF-α and IL-6 and inhibited MH-S cell viability. SNHG14 silencing or miR-34c-3p over-expression decreased the wet/dry weight ratio in lung tissues from ALI mice. The reductions induced by SNHG14 silencing or miR-34c-3p over-expression were rescued by WISP1 over-expression.

**Conclusion:**

This study demonstrated that lncRNA SNHG14 silencing alleviated inflammation in LPS-induced ALI through miR-34c-3p-mediated inhibition of WISP1*.* Our findings suggest that lncRNA SNHG14 may serve as a therapeutic target for ALI.

## Introduction

Acute lung injury (ALI) is a milder form of acute respiratory distress syndrome (ARDS), caused by numerous conditions, such as pneumonia, sepsis, and trauma [1]. ALI is associated with respiratory failure that leads to significant morbidity and sometimes even death [2]. The diagnosis of ALI relies on clinical and radiographic criteria, which are sometimes non-specific, resulting in inaccurate diagnoses [3]. Therefore, the complete understanding of the pathologies and molecular mechanisms of ALI is of great importance in improving the diagnosis and treatment of patients with ALI.

Lipopolysaccharide (LPS) is located in the outer membrane’s outer leaflet of many gram-negative bacteria and regarded as a well-characterized pathogen, which is linked with molecular pattern [4]. In addition, LPS also serves as a mediator of pro-inflammation and displays essential characteristics of microvascular lung injury reproducibly when delivered intratracheally [5]. Alveolar macrophages differ from macrophages that are located between the airway epithelium and blood vessels, indicating that macrophage populations are subjected to further specialization within the lungs; and alveolar macrophages are present in tissue compartments, which further suggests that significant fluctuations of environment can lead to differentiation [6]. Strikingly, macrophages such as MH-S cells have been recognized to be fundamental to the inflammatory response induced by ALI in addition to leukocytes and endothelial cells. Several studies have also highlighted that regulating their functions can be used as a potential therapeutic option against ALI [7, 8]. Herein, the current study aims to elucidate the functions of mouse alveolar macrophages (MH-S) exposed to LPS for induction of ALI.

Furthermore, long non-coding RNAs (lncRNAs), a relatively new class of heterogeneous non-coding RNAs, have been found in numerous species [9]. LncRNAs play essential roles in biological processes, and as a result lncRNA dysfunctions or dysregulations are linked to various diseases [10]. In addition, lncRNAs are involved in regulating gene expression in multiple ways, including chromosome remodeling and transcriptional and post-transcriptional processing [11]. Down-regulated lncRNA small nucleolar RNA host gene 14 (SNHG14) was previously demonstrated to inhibit the proliferation of non-small cell lung cancer (NSCLC) by inducing cell arrest and apoptosis [12]. Up-regulation of SNHG14, on the other hand, has been recognized to aggravate inflammation in cerebral ischemia/reperfusion injury by impairing the miR-136-5p-dependent inhibition of ROCK1 [13]. Importantly, bioinformatics analysis available at lncRNABase website revealed that SNHG14 might bind to miR-34c-3p. MiRNAs are pivotal gene expression regulators, and consequently have significant roles in several biological processes, including cell metabolism, proliferation, differentiation, and apoptosis [14]. In LPS-treated periodontal ligament cells, miR-34c-3p were identified as a down-regulated miRNA in periodontitis, a chronic inflammatory disease [15]. Moreover, bioinformatics analysis available at microRNA.org website in the early phases of our investigation revealed the existence of possible binding sites between miR-34c-3p and Wnt1-inducible signaling pathway protein-1 (WISP1). WISP1, also known as CNN4, belongs to the CCN family and possess the ability to regulate cell growth, transformation and survival in a tissue-specific manner [16]. Another previous study demonstrated that suppression of the WISP1-integrin β6 pathway alleviated ALI in a mice model of sepsis [17]. Based on these previous findings, we therefore hypothesize that there is a high possibility that SNHG14, miR-34c-3p, and WISP1 may interact with each other and are implicated in the pathogenesis and progression of ALI.

## Materials and methods

### Ethics statement

All experimental protocols in the current study were approved by the Ningxia Medical University General Hospital Scientific Research Ethics Committee. Animal related procedures were performed according to the recommendations in the Guide for the Care and Use of Laboratory Animals (8th Edition, 2011, National Research Council).

### ALI cell model in vitro

MH-S cells (Cell resource Center, Chinese Academy of Medical Sciences & Peking Union Medical College, Beijing, China) were cultured in Ham’s F-12 K Medium containing 1.59/L sodium bicarbonate, 15% fetal bovine serum and 2 mM L-glutamine in a humidified incubator with 5% CO_2_ in air at 37 °C. This culture solution was renewed every 2–3 days.

Cells were inoculated in a 96-well culture plate at a density of 4 × 10^5^ cells/mL in 100 μL for 1 h. Next, the 12 K medium was then added to the control group. Except for the blank group, LPS (100 μg/mL) was added to the remaining groups to establish ALI cell models. Subsequently, the cells were transfected using a Lipofectamine 2000 kit (Invitrogen, Thermo Fisher Scientific, Waltham, MA, USA).

### ALI mouse model in vivo

Mice obtained from the Institute of Cancer Research (*n* = 80, 18–24 g, Hunan SJA Laboratory Animal Co., Ltd., Hunan, China; SCXK, Xiang, 2009–0004) were maintained under specific pathogen-free conditions. The mice were then randomly assigned into the control (*n* = 10) and ALI (*n* = 70) groups and intraperitoneally anesthetized with 3% amobarbital (100 mg/kg). Mice were placed in a supine position to prepare for subsequent surgical procedures. A 0.5 cm longitudinal incision was made at the neck approximately 1–1.5 cm below the teeth. Subcutaneous tissues were carefully separated to expose the trachea. LPS diluted in 300 μL sterile normal saline was injected into the trachea of mice in the ALI group, whereas the mice in the control group received an injection of 300 μL sterile normal saline. Six hours after LPS administration, the mice were grouped (*n* = 10 mice/group). MH-S cells (1 × 10^6^ cells, 500 μL) infected with adenovirus harboring SNHG14-antisense oligonucleotide (SNHG14-ASO), miR-34c-3p mimic or over-expressed WISP1 (oe-WISP1) (Zhonghong Boyuan Biological Technology Co., Ltd., Shenzhen, China) were injected into the mice via the caudal vein. After 48 h, the trachea was cannulated, the lungs were rinsed with 1.2 mL phosphate-buffered saline (PBS) 3 times and the solution was collected. Mice were then euthanized and the degree of lung injury was observed.

### Subcellular localization of SNHG14 determined by fluorescence in situ hybridization (FISH)

Ribo™ lncRNA FISH Probe Mix (red, C10920, Guangzhou RiboBio Co., Ltd., Guangzhou, Guangdong, China) was employed in the current study. Cells (6 × 10^4^ cells/well) were seeded in a 24-well plate and fixed with 1 mL of 4% paraformaldehyde for 10 min. Pre-cooled PBS containing 0.5% Triton X-100 (1 mL) was added to each well and allowed to stand at 4 °C for 5 min. Next, the pre-hybridization solution (200 μL) was added and sealed at 37 °C for 30 min. After removal of the pre-hybridization solution, the hybridization solution containing anti-SNHG14 oligonucleotide probes (Wuhan GeneCreate Biological Engineering Co., Ltd., Wuhan, Hubei, China) was added to each well and allowed to hybridize overnight at 37 °C in dark conditions. On the following day, the cells were washed with cleansing solution I at 42 °C (4 × saline sodium citrate (SSC), 0.1% Tween-20), cleansing solution II (2 × SSC), cleansing solution III (1 × SSC) and 1 × PBS successively. Cells were then stained with 4′,6-diamidino-2-phenylindole at a ratio of 1: 800 for 10 min and mounted with nail polish. Finally, the cells were observed and imaged in 5 random fields under a fluorescence microscope (Olympus, Tokyo, Japan).

### Dual-luciferase reporter assay

The lncRNABase (http://starbase.sysu.edu.cn/mirLncRNA.php) was applied to predict the downstream miRNAs that could be regulated by SNHG14. In addition, the target genes of miR-34c-3p were analyzed using the online website, microRNA.org. Based on the predicted binding sites, wild-type (wt) SNHG14 sequence and mutant (mut) sequence, as well as WISP1 mRNA 3′-untranslated region (3′-UTR) sequences and mutant sequences were synthesized and introduced into the pmirGLO luciferase vectors (E1330, Promega, Madison, WI, USA) to generate wt-SNHG14, mut-SNHG14, wt-WISP1, and mut-WISP1 plasmids (GenePharma Co., Ltd., Shanghai, China). Negative control (NC) mimic or miR-34c-3p mimic was co-transfected with wt-SNHG14 (containing ACACACCAATCACTA that bound to miR-34c-3p), mut-SNHG14 (in absence of ACACACCAATCACTA that bound to miR-34c-3p), wt-WISP1 (containing TCACTA that bound to miR-34c-3p) and mut-WISP1 (in absence of TCACTA that bound to miR-34c-3p) into 293 T cells for 48 h. Dual-luciferase reporter assay kits (D0010, Beijing Solarbio Science & Technology Co., Ltd., Beijing, China) and GLomax20/20 luminometer (E5311, Shaanxi Zhongmei Biotechnology Co., Ltd., Shaanxi, China) were used to determine the luciferase activity.

### RNA binding protein immunoprecipitation (RIP)

RIP lysis buffer (N653–100 mL, Shanghai Haoran Biological Technology Co., Ltd., Shanghai, China) was added to the MH-S cells and maintained on ice for 5 min to prepare the cell lysate. Magnetic beads (50 μL) and RIP wash buffer (0.5 mL, EHJ-BVIS08102, Xiamen Huijia Biotechnology Co., Ltd., Xiamen, China) were added to each sample tube. After brief mixing, the tubes were placed on a magnetic separator until the beads were collected. RIP wash buffer (100 μL) was added to re-suspend the magnetic beads followed by the addition of 5 μg of Argonaute2 (Ago2) antibody (P10502500, Otwo Biotech Inc., Shenzhen, China). Immunoglobulin G (IgG) was added instead of the Ago2 antibody as NC. Beads were then washed twice with 0.5 mL RIP wash buffer. Next, the RIP Immunoprecipitation buffer (900 μL, P10403138, Otwo Biotech Inc., Shenzhen, China) was added to the bead-antibody mixture and centrifuged at 14,000 rpm at 4 °C for 10 min. The supernatant was then collected and transferred into new Eppendorf tubes (LBCT015S, Beijing North TZ-Biotech Develop, Co., Ltd., Beijing, China). The mixture (1 mL) was incubated overnight at 4 °C with mild rotation to allow uniform mixing of the contents. Beads were then washed with 0.5 mL RIP wash buffer for 6 times. Proteinase K buffer (150 μL) was added and incubated at 55 °C for 30 min to purify the RNA. Total RNA content was extracted using the Trizol method and subjected to reverse transcription quantitative polymerase chain reaction (RT-qPCR).

### Enzyme-linked immunosorbent assay (ELISA)

Cell concentration was adjusted to 4 × 10^5^ cells/mL and seeded (500 μL/well) in a 24-well plate and allowed to culture for 1 h. LPS was added to each well and cultured for another 24 h. Cells were then centrifuged at 1500 rpm for 10 min and the supernatant was collected for ELISA.

Bronchoalveolar lavage fluid from ALI mice was stored at 4 °C and centrifuged at 3000 rpm for 10 min. The supernatant was then collected and stored at − 20 °C for determination of cytokines; interleukin (IL)-18, IL-1β, tumor necrosis factor (TNF)-α and IL-6 using ELISA kits (Multi Science, Hangzhou, China).

### Cell counting kit (CCK)-8 assay

MH-S cells were seeded in a 96-well plate containing 2 × 10^3^ cells/well after transfection for 24 h, 48 h or 72 h and cultured for an additional 16 h. The culture medium was then replaced by freshly prepared 100 μL culture medium containing 10% CCK-8 and incubated for 1 h. The absorbance value of each well was analyzed with a microplate reader at a wavelength of 450 nm.

### Determination of pulmonary edema

The left lung that was not subjected to bronchoalveolar lavage was collected by means of thoracotomy. The wet (W) weight was recorded and dry (D) weight was measured, after the lung was baked at 80 °C for 48 h. The W/D weight ratio was then calculated using the following formula: wet weight/dry weight × 100%.

### Morphological changes in lung tissues determined by hematoxylin-eosin (HE) staining

The right side of the lung without bronchoalveolar lavage was fixed with formalin solution and prepared into 5 μm sections. Sections were then baked at 60 °C for 1 h and dewaxed by xylene. After hydration, the sections were stained by HE (Beijing Solarbio Science & Technology Co., Ltd., Beijing, China), then dehydrated with gradient alcohol, cleared by xylene and mounted with neutral gum. Lung morphological changes were observed under an optical microscope (XP-330, Shanghai Bingyu Optical Instrument Co., Ltd., Shanghai, China).

### Measurement of myeloperoxidase (MPO) activity

Lung tissue homogenates were mixed with thiobarbituric acid (TBA). The mixture was centrifuged and the absorbance per gram of lung tissue was detected in the supernatant using spectrophotometry to evaluate the MPO activity.

### Wright-Giemsa staining

The extracted bronchoalveolar lavage fluid (BALF) was dispersed into a single cell suspension, and the number of cells was counted under a microscope. Then, 15 mL of the cell suspension was fixed in methanol for 2–3 min, and stained with diluted Wright-Giemsa staining solution (Shanghai Rongbai Biological Technology Co., Ltd., Shanghai, China) for 15–30 min at room temperature.

### RT-qPCR

Total RNA content was extracted using Trizol reagent kits. Primers used are presented in Table 1 (Takara Bio Inc., Tokyo, Japan). Total RNA was reversely transcribed into complementary DNA (cDNA) using PrimeScript RT reagent kits (RRO36A, Takara Biotechnology Ltd., Dalian, Liaoning, China). A SYBR® Premix Ex TaqTM II reagent kit (RR820A, Takara) was applied to perform RT-qPCR with an ABI7500 real-time qPCR system (7500, ABI Company, Oyster Bay, NY, USA). U6 was regarded as the internal reference for miR-34c-3p. Glyceraldehyde-3-phosphate dehydrogenase (GAPDH) was used as an internal reference for SNHG14 and WISP1. Relative quantification was calculated using the 2^-△△CT^ method.

**Table 1:** Primer sequences for RT-qPCR

	Sequence (5′-3′)
SNHG14	F: ACCTGCAAGCTTTTTGACCC
	R: AGCAGACAAAGAAAAACCCCAAT
miR-34c-3p	F: GCCCAATCACTAACCACACGG
	R: GTGCAGGGTCCGAGGT
WISP1	F: ACCACCTGTGGCCTAGGTAT
	R: CCTGCGAGAGTGAAGTTCGT
U6	F: GTGATCACTCCCTGCCTGAG
	R: GGACTTCACTGGACCAGACG
GAPDH	F: CCGCATCTTCTTGTGCAGTG
	R: CCCAATACGGCCAAATCCGT

*RT-qPCR* Reverse transcription quantitative polymerase chain reaction, *SNHG14* Small nucleolar RNA host gene 14, *miR* MicroRNA, *WISP1* Wnt1-inducible signaling pathway protein 1, *GAPDH* Glyceraldehyde-3-phosphate dehydrogenase, *F* Forward, *R* Reverse

### Western blot analysis

Total protein content in tissues or cells was extracted by radio-immunoprecipitation assay lysis buffer containing phenylmethylsulfonyl fluoride. Protein concentration was determined by a bicinchoninic acid kit. Next, the proteins were separated by sodium dodecyl sulfate-polyacrylamide gel electrophoresis and transferred onto a polyvinylidene fluoride membrane. Membranes were then blocked with 5% skim milk powder for 1 h at room temperature and incubated with the primary antibodies (Abcam Inc., Cambridge, MA, USA) of rabbit anti-mouse antibodies to WISP1 (ab178547, dilution ratio of 0.5 μg/mL), caspase-1 (ab1872, dilution ratio of 1: 1000) and GAPDH (ab9485, dilution ratio of 1: 2500, internal reference) overnight at 4 °C. Membranes were then washed with Tris-buffered saline Tween-20 and further incubated with the horseradish peroxidase-conjugated secondary antibody of goat anti-rabbit IgG (ab97051, dilution ratio of 1: 2000; Abcam Inc., Shanghai, China) for 1 h at room temperature. Proteins on the membrane were visualized by enhanced chemiluminescence detection kits (BB-3501, Amersham Pharmacia Biotech, UK) and Bio-Rad image analysis system (Bio-Rad Laboratories, Inc. CA, USA). The protein band intensity was determined using the Quantity One v4.6.2 software. The ratio of gray value of target protein band to that of GAPDH was regarded as the relative protein expression.

### Statistical analysis

Statistical analyses were carried out using the SPSS 21.0 software (IBM Corp., Armonk, NY, USA). Measurement data were expressed as mean ± standard deviation. Comparisons between two groups were analyzed using the unpaired *t-*test. Cell viability at the 24th h, 48th h and 72nd h was compared by two-way analysis of variance (ANOVA) with non-repeated measure. Pearson’s correlation was applied to analyze the correlation between miR-34c-3p and lncRNA SNHG14 expression. A value of of *p* < 0.05 were considered to be statistically significant.

## Results

### LncRNA SNHG14 is highly expressed in LPS-treated cell and mouse ALI models

**Fig. 1:**
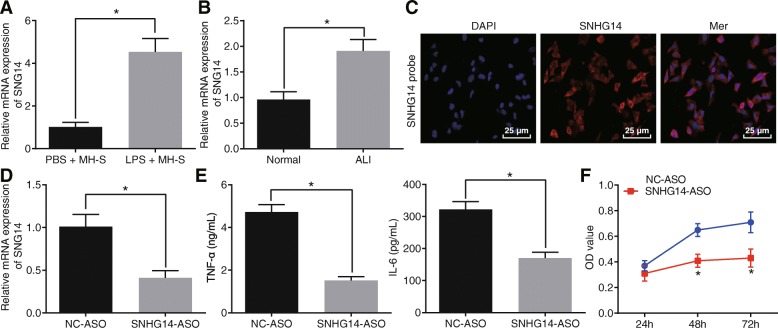
LncRNA SNHG14 is up-regulated in ALI models and its knockdown protects against LPS-induced inflammation. A, expression of lncRNA SNHG14 in MH-S cells treated with LPS or PBS determined by RT-qPCR, * *p* < 0.05 vs. the PBS + MH-S group (MH-S treated with PBS), *n* = 10. B, expression of lncRNA SNHG14 in lung tissues from mice with ALI induced by LPS or mice treated with normal saline determined by RT-qPCR, * *p* < 0.05 vs. the normal group (mice treated with normal saline), *n* = 10. C, representative micrographs showing localization of lncRNA SNHG14 in MH-S cells identified by FISH (400 ×). D, expression of lncRNA SNHG14 after SNHG14-ASO transfection determined by RT-qPCR, * *p* < 0.05 vs. the NC-ASO group (MH-S cells transfected with NC-ASO). E, TNF-α and IL-6 levels in the MH-S cell supernatant after lncRNA SNHG14 silencing determined by ELISA, * *p* < 0.05 vs. the NC-ASO group (MH-S cells transfected with NC-ASO). F, the MH-S cell viability after lncRNA SNHG14 silencing measured by CCK-8 assay, * *p* < 0.05 vs. the NC-ASO group (MH-S cells transfected with NC-ASO). The statistical values were measurement data and expressed as mean ± standard deviation. Unpaired *t*-test was used for comparisons between two groups. Statistical analysis in relation to time-based measurements within each group was realized using two-way ANOVA with non-repeated measure. The experiment was repeated 3 times independently

SNHG14 expression was higher in MH-S cells treated with LPS in comparison with the control cells (Fig. 1a). Similarly, SNHG14 was also found to be higher in lung tissues from mice with LPS-induced ALI versus the control mice (Fig. 1b).

Subcellular localization of SNHG14 in MH-S cells was determined using FISH. SNHG14 was found to be primarily expressed in the cytoplasm (Fig. 1c). Subsequently, SNHG14 expression was effectively reduced in MH-S cells by transfection with SNHG14-ASO (Fig. 1d).

On the other hand, pro-inflammatory cytokines were determined in MH-S cell supernatant following SNHG14 knock-down by ELISA. SNHG14 silencing decreased TNF-α and IL-6 levels (Fig. 1e). In addition, CCK-8 assay demonstrated that SNHG14 silencing also decreased MH-S cell viability (Fig. 1f). Taken together, these results showed that SNHG14 was up-regulated in ALI, while SNHG14 silencing may alleviate the inflammatory response in ALI.

### miR-34c-3p expression is reduced in LPS-exposed MH-S cells and its up-regulation inhibits LPS-induced acute inflammatory injury

**Fig. 2:**
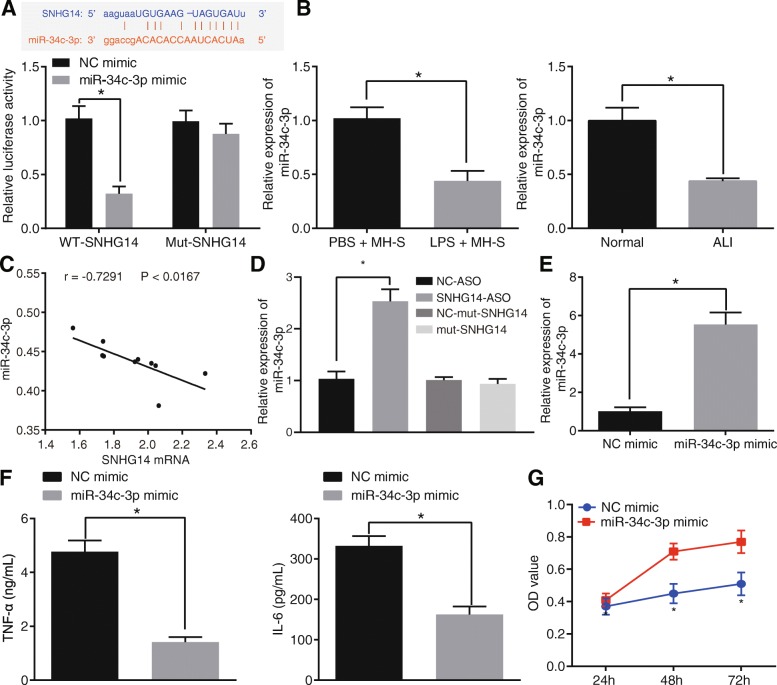
miR-34c-3p is down-regulated in ALI and its over-expression protects against LPS-induced inflammation. A, the binding relationship between miR-34c-3p and lncRNA SNHG14 determined by online software (upper panel) and dual-luciferase reporter assay (lower panel), * *p* < 0.05 vs. the NC mimic group (MH-S cells transfected with NC mimic). B, miR-34c-3p expression in MH-S cells treated with LPS and mice with LPS-induced ALI determined by RT-qPCR, * *p* < 0.05 vs. the PBS + MH-S group (MH-S cells treated with PBS), *n* = 10. C, Pearson correlation analysis of miR-34c-3p and lncRNA SNHG14 expression. D, the effect of lncRNA SNHG14 silencing on the expression of miR-34c-3p detected by RT-qPCR, * *p* < 0.05 vs. the NC-ASO group (MH-S cells transfected with NC-ASO). E, the transfection efficiency of miR-34c-3p mimic detected by RT-qPCR, * *p* < 0.05 vs. the NC mimic group (MH-S cells transfected with NC mimic). F, the levels of TNF-α and IL-6 in the MH-S cell supernatant were determined by ELISA. * *p* < 0.05 vs. the NC mimic group (MH-S cells transfected with NC mimic). G, MH-S cell viability in response to miR-34c-3p mimic transfection assessed by CCK-8 assay, * *p* < 0.05 vs. the NC mimic group (MH-S cells transfected with NC mimic). The statistical values were measurement data and expressed as mean ± standard deviation. Comparison between two groups was analyzed using unpaired *t-*test. Statistical analysis in relation to time-based measurements within each group was realized using two-way ANOVA with non-repeated measure. The experiment was repeated 3 times independently

Initial online analysis revealed the existence of a binding site between SNHG14 and miR-34c-3p (upper panel, Fig. 2a), and subsequently, a dual-luciferase reporter assay was applied to determine this relationship. Luciferase activity was found to be reduced after co-transfection with wt-SNHG14 and miR-34c-3p mimic as compared to the NC mimic (lower panel, Fig. 2a). However, luciferase activity did not vary in cells co-transfected with mut-SNHG14 and miR-34c-3p mimic. These findings suggested a binding relationship between SNHG14 and miR-34c-3p.

Additionally, decreased miR-34c-3p expression was observed in MH-S cells treated with LPS as well as in mice with LPS-induced ALI (Fig. 2b). Pearson’s analysis revealed an inverse relationship between SNHG14 and miR-34c-3p expression, wherein increased SNHG14 resulted in a decrease in the expression of miR-34c-3p (Fig. 2c). Furthermore, SNHG14 silencing significantly increased the expression of miR-34c-3p, while no significant differences were detected upon transfection with mut-SNHG14 (Fig. 2d). Therefore, these results demonstrated that SNHG14 bound to and negatively-regulated the expression of miR-34c-3p.

On the other hand, miR-34c-3p mimic-transfected MH-S cells were used to determine the effects of miR-34c-3p on pro-inflammatory cytokines. As expected, miR-34c-3p expression was elevated after miR-34c-3p mimic transfection (Fig. 2e). In addition, the levels of TNF-α and IL-6 in MH-S cell supernatant were measured using ELISA. The results revealed that miR-34c-3p mimic significantly increased TNF-α and IL-6 levels (Fig. 2f). Meanwhile, miR-34c-3p mimic transfection resulted in a decrease in MH-S cell viability (Fig. 2g). Taken together, miR-34c-3p was expressed at low-levels in ALI, and over-expression of miR-34c-3p may help alleviate inflammatory reactions during ALI.

### LncRNA SNHG14 binds to miR-34c-3p to up-regulate WISP1 expression

**Fig. 3:**
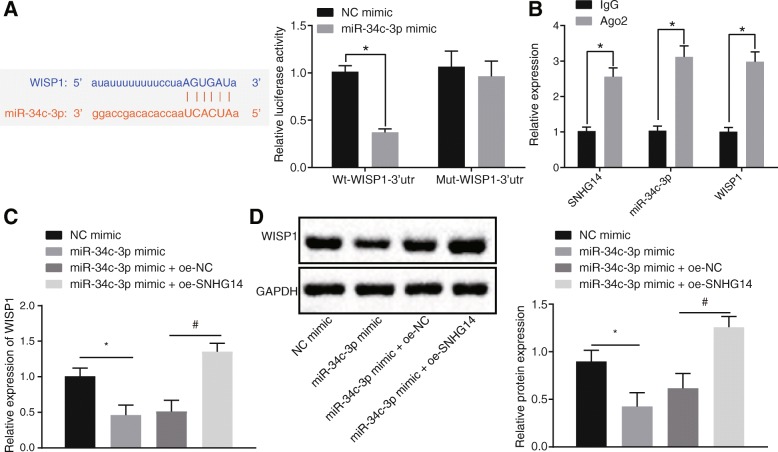
LncRNA SNHG14 and WISP1 bind to miR-34c-3p. A, the binding of miR-34c-3p to WISP1 was confirmed by dual-luciferase reporter assay, * *p* < 0.05 vs. the NC mimic group (cells transfected with NC mimic). B, interaction between miR-34c-3p and lncRNA SNHG14 or WISP1 determined by RIP assay. * *p* < 0.05 vs. the anti-IgG group (MH-S cells incubated with anti-IgG). C, mRNA expression of WISP1 after transfection with miR-34c-3p mimic or miR-34c-3p mimic and oe-SNHG14 determined by RT-qPCR, * *p* < 0.05 vs. the NC mimic group (MH-S cells transfected with NC mimic), # *p* < 0.05 vs. the miR-34c-3p mimic + oe-NC group (MH-S cells co-transfected with miR-34c-3p mimic and oe-NC). D, Western blot analysis of WISP1 protein expression normalized to GAPDH after transfection with miR-34c-3p mimic or miR-34c-3p mimic and oe-SNHG14, * *p* < 0.05 vs. the NC mimic group (MH-S cells transfected with NC mimic), # *p* < 0.05 vs. the miR-34c-3p mimic + oe-NC group (MH-S cells co-transfected with miR-34c-3p mimic and oe-NC). The statistical values were measurement data and expressed as mean ± standard deviation. Comparison was analyzed using unpaired *t-*test in panels A and B or using one-way ANOVA in panels C and D. The experiment was repeated 3 times independently

The binding site between miR-34c-3p and WISP1 was originally detected using website analysis (Left panel, Fig. 3a). This allowed us to further analyze whether miR-34c-3p could target and regulate WISP1 to mediate the inflammatory responses in ALI.

We further conducted a dual-luciferase reporter assay, the results of which showed that miR-34c-3p mimic markedly reduced the luciferase activity of wt-WISP1 (*p* < 0.05), but no effects on that of mut-WISP1 (Fig. 3a), which exceedingly suggests the binding of miR-34c-3p to WISP1. In addition, RIP assay showed that anti-Ago2 group exhibited increased expression of SNHG14 or WISP1 binding to miR-34c-3p in MH-S cells when compared to anti-IgG group (Fig. 3b). Moreover, miR-34c-3p mimic transfection decreased the mRNA and protein expression of WISP1 mRNA (Fig. 3c, Fig. 3d), while over-expression SNHG14 neutralized the effects of miR-34c-3p. These findings suggested that SNHG14 and WISP1 could competitively bind to miR-34c-3p. Reducing the expression of SNHG14 led to enhanced binding of miR-34c-3p to WISP1, and in turn decreased the WISP1 expression.

### Down-regulated lncRNA SNHG14 alleviates LPS-induced ALI via miR-34c-3p-mediated WISP1 inhibition in vitro

**Fig. 4:**
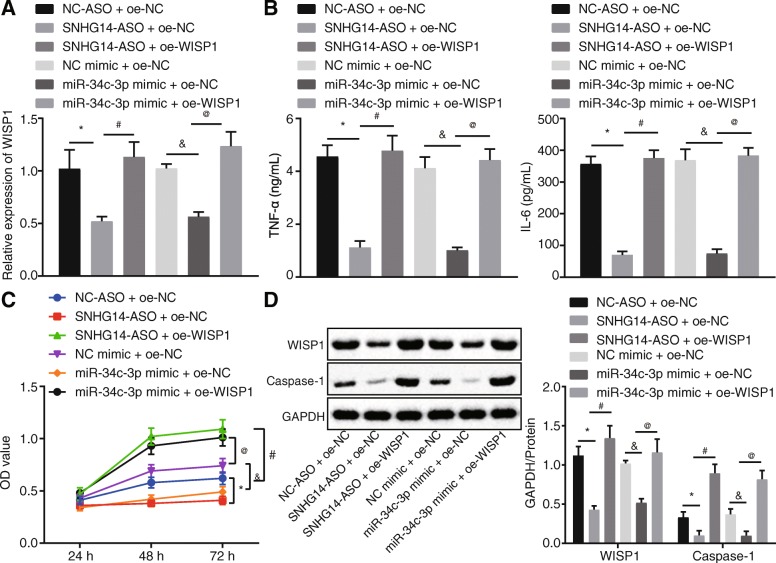
Down-regulated lncRNA SNHG14 prevents LPS-induced ALI via miR-34c-3p-mediated WISP1 inhibition in vitro. MH-S cells were transfected with miR-34c-3p mimic, SNHG14-ASO, or co-transfected with SNHG14-ASO and oe-WISP1, or miR-34c-3p mimic and oe-WISP1. A, mRNA expression of WISP1 after transfection determined by RT-qPCR. B, the levels of TNF-α and IL-6 in MH-S cell supernatant after transfection measured by ELISA. C, the MH-S cell viability determined by CCK-8 assay, D, Western blot analysis of WISP1 and caspase-1 protein normalized to GAPDH in MH-S cells after transfection, * *p* < 0.05 vs. the NC-ASO + oe-NC group (MH-S cells co-transfected with NC-ASO + oe-NC), # *p* < 0.05 vs. the SNHG14-ASO + oe-NC group (MH-S cells co-transfected with SNHG14-ASO + oe-NC), & *p* < 0.05 vs. the NC mimic + oe-NC group (MH-S cells co-transfected with NC mimic + oe-NC), @ *p* < 0.05 vs. the miR-34c-3p mimic + oe-NC group (MH-S cells co-transfected with miR-34c-3p mimic + oe-NC). The statistical values were measurement data and expressed as mean ± standard deviation. Comparison was analyzed using one-way ANOVA in panels A, B and D and using two-way ANOVA with non-repeated measure in panel C. The experiment was repeated 3 times independently

MH-S exposed to LPS were co-treated with NC-ASO and oe-NC, SNHG14-ASO and oe-NC, SNHG14-ASO and oe-WISP1, NC mimic and oe-NC, miR-34c-3p mimic and oe-NC or miR-34c-3p mimic and oe-WISP1. WISP1 mRNA expression was increased in response to SNHG14-ASO + oe-WISP1 treatment when compared with SNHG14-ASO and oe-NC treatment. In addition, WISP1 mRNA expression was increased upon miR-34c-3p mimic and oe-WISP1 treatment than those subjected to miR-34c-3p mimic and oe-NC treatment (Fig. 4a).

According to the results of ELISA, TNF-α and IL-6 levels in the MH-S cell supernatant inhibited by either SNHG14-ASO or miR-34c-3p mimic were rescued by oe-WISP1 transfection (Fig. 4b).

In addition, cell viability was measured using CCK-8 assay. The results revealed that the viability of cells co-transfected with SNHG14-ASO and oe-WISP1 was significantly higher compared to that of cells transfected with SNHG14-ASO and oe-NC (Fig. 4c). Cell viability was also increased in response to co-transfection with miR-34c-3p mimic and oe-WISP1 when compared with that of cells co-transfected with miR-34c-3p mimic and oe-NC (Fig. 4c).

Protein expression of WISP1 and caspase-1 was increased in response to SNHG14-ASO and oe-WISP1 co-transfection versus SNHG14-ASO and oe-NC co-transfection. Similarly, miR-34c-3p mimic and oe-WISP1 co-transfection led to increased WISP and caspase-1 protein expression when compared to miR-34c-3p mimic and oe-NC co-transfection (Fig. 4d). Taken together, these results demonstrated that SNHG14 bound to miR-34c-3p to up-regulate WISP1. On the other hand, SNHG14 silencing increased the binding of miR-34c-3p to WISP1 and inhibited WISP1, leading to reduced inflammation in ALI in vitro*.*

### Down-regulated lncRNA SNHG14 alleviates LPS-induced ALI via miR-34c-3p-mediated WISP1 inhibition in vivo

**Fig. 5:**
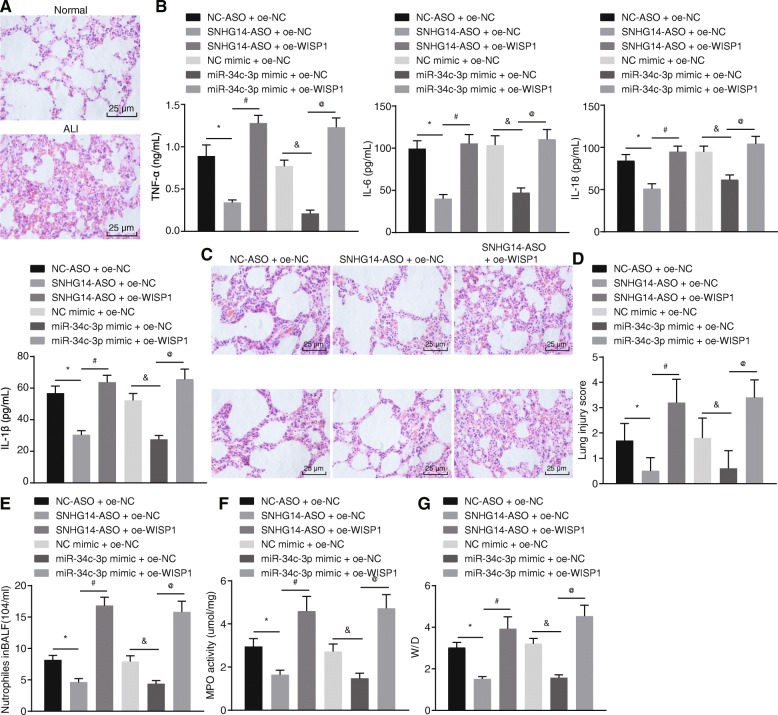
Down-regulated lncRNA SNHG14 prevents LPS-induced ALI via miR-34c-3p-mediated WISP1 inhibition in vivo. A, the representative micrographs showing lung tissue morphological changes after HE staining (400 ×), *n* = 10. B, the levels of IL-18, IL-1, TNF-α, and IL-6 in bronchoalveolar lavage fluid measured by ELISA, *n* = 10. C, representative images showing pathological changes in lung tissues from ALI mice injected with adenoviruses harboring different plasmids observed after HE staining (400 ×), *n* = 10. D, lung injury score of ALI mice injected with adenoviruses harboring different plasmids. E, the neutrophils in the BALF of ALI mice injected with adenoviruses harboring different plasmids. F, MPO activity in ALI mice injected with adenoviruses harboring different plasmids. G, pulmonary edema evaluated by determining wet/dry weight ratio in ALI mice injected with adenoviruses harboring different plasmids, *n* = 10. * *p* < 0.05 vs. the NC-ASO + oe-NC group, # *p* < 0.05 vs. the SNHG14-ASO + oe-NC group, & *p* < 0.05 vs. the NC mimic + oe-NC group, @ *p* < 0.05 vs. the miR-34c-3p mimic + oe-NC group. The statistical values were measurement data and expressed as mean ± standard deviation. Comparisons were analyzed using one-way ANOVA. The experiment was repeated 3 times independently

Microscopic observation revealed that the alveolar structure in the lung from normal mice was intact without thickening or lymphocyte infiltration (left panel, Fig. 5a). However, pulmonary lesions in LPS-treated mice were evident, which presented with pathologically thickened alveolar walls, collapsed alveoli and numerous infiltrated red blood cells and inflammatory cells (right panel, Fig. 5a), indicating that our ALI mice models were successfully established.

Levels of inflammatory factors, IL-18, IL-1, TNF-α and IL-6 in bronchoalveolar lavage fluid were determined to be significantly higher in LPS-treated mice injected with adenoviruses harboring SNHG14-ASO and oe-WISP1 as compared to those in LPS-treated mice injected with adenoviruses harboring SNHG14-ASO and oe-NC (Fig. 5b). In addition, LPS-treated mice injected with miR-34c-3p mimic and oe-WISP1 exhibited markedly increased levels of these inflammatory cytokines when compared to those injected with miR-34c-3p mimic and oe-NC. As shown in Fig. 5c-d after HE staining, aggravated lung tissue damage, alveolar wall thickening, increased alveolar collapse, red blood cell and inflammatory cell infiltration as well as higher lung injury score were observed in LPS-treated mice injected with adenoviruses harboring SNHG14-ASO and oe-WISP1 as compared to those in LPS-treated mice injected with adenoviruses harboring SNHG14-ASO and oe-NC. The above-mentioned features of aggravated lung tissue damage were also observed in LPS-treated mice when miR-34c-3p and WISP1 were simultaneously up-regulated as compared to those in LPS-treated mice when only miR-34c-3p was upregulated. Next, measurement of MPO activity and Wright-Giemsa staining were employed to evaluate neutrophil infiltration in lung tissues and BALF. The data displayed that reduced MPO activity and neutrophil infiltration caused by either silencing of SNHG14 or upregulation of miR-34c-3p were rescued by over-expression of WISP1 (Fig. 5e-f). The W/D weight ratio (edema formation) was also calculated to be increased in LPS-treated mice injected with adenoviruses harboring SNHG14-ASO and oe-WISP1 when compared with LPS-treated mice injected with adenoviruses harboring SNHG14-ASO and oe-NC. Similarly, LPS-treated mice injected with adenoviruses harboring miR-34c-3p mimic and oe-WISP1 exhibited increased W/D weight ratio when compared with LPS-treated mice injected with adenoviruses harboring miR-34c-3p mimic and oe-NC (Fig. 5g). These findings suggested that SNHG14 was able to bind to miR-34c-3p and thus, up-regulate WISP1. SNHG14 silencing increased the binding of miR-34c-3p to WISP1 and down-regulated WISP1, leading to reduced inflammation in ALI in vivo*.*

## Discussion

ALI is defined as a syndrome of acute inflammation and increased permeability in the alveolar-capillary membrane associated with at least one risk factor [18]. Notably, miRNAs have been indicated to play a key role in the regulation of genes implicated in the process of acute inflammatory lung injury [19]. In this study, we found that miR-34c-3p was targeted and down-regulated by lncRNA SNHG14, leading to increased WISP1 in both cell and mice models of LPS-induced ALI. On the contrary, lncRNA SNHG14 silencing resulted in an up-regulation of miR-34c-3p and subsequent down-regulation of WISP1 expression, leading to reduced ALI severity.

Initially, we uncovered that LPS-exposed MH-S cells and LPS-injured lung tissues of mice exhibit high expression of lncRNA SNHG14. Similarly, up-regulated levels of SNHG14 have also been documented in oxygen-lacking conditions such as in cerebral ischemic animals and/or oxygen-glucose deprived cells [20]. Moreover, another study has reported that elevated expression of lncRNA SNHG14 in ischemic brain tissues contributes to inflammatory response and neurological impairment, which is partially in line with our findings [13]. Our results and findings suggest the use of SNHG14 silencing to prevent LPS-induced inflammation, thereby protecting against ALI. Previous studies have also echoed our sentiment statement that down-regulation of lncRNA MALAT1 suppresses the inflammatory response in ALI rats [21]. Additionally, SNHG14 knockdown suppresses the activation of microglia cells which release inflammatory cytokines following ischemic stroke, in so doing conferring protection against cerebral infarction [22]. Our results also indicate towards a similar regulation, wherein SNHG14 silencing led to a decrease in the viability of MH-S cells and reduction in the release of inflammatory proteins. Another significant finding was that the expression of miR-34c-3p was reduced in LPS-induced ALI tissues. Moreover, over-expression of miR-34c-3p was previously demonstrated to inhibit the progression of LPS-induced ALI. Available data also suggests that miRNAs are involved in polarization of macrophages bearing critical roles in ALI/ARDS pathogenesis [23]. MiR-34c-3p also exhibits down-regulated expression in the lung tissues of silica-exposed rats, which may be involved in the process of pulmonary fibrosis in early silicosis [24]. Our study evidenced that miR-34c-3p suppresses LPS-induce inflammation and viability of MH-S cells to alleviate ALI. Given the above-mentioned findings, the involvement of lncRNA SNHG14 and miR-34c-3p in the pathogenesis of ALI is validated.

Additionally, mechanistic analyses demonstrated that lncRNA SNHG14 upregulates the WISP1 gene via binding to miR-34c-3p. WISP1 is not only expressed in multiple different tissues [16], but also up-regulated in ventilator-induced lung injury models [25]. Moreover, recent reports have indicated that WISP1 is regulated by other miRNAs. For instance, miR-92a targets and negatively-regulates WISP1, which prevents the development of idiopathic pulmonary fibrosis [26]. Similar regulatory mechanisms have also been identified, wherein lncRNA SNHG14 binds to miR-193a-3p in breast cancer, and silencing of lncRNA SNHG14 up-regulates miR-193a-3p to inhibit the growth and invasion of breast cancer cells [27]. Besides, lncRNA SNHG14 also down-regulates miR-101 to facilitate cell progression in pancreatic ductal adenocarcinoma, while we discovered that lncRNA SNHG14 performs a likewise role in ALI [28]. Furthermore, our findings established that both lncRNA SNHG14 silencing or miR-34c-3p over-expression are capable of reducing the levels of pro-inflammatory cytokines (IL-18, IL-1, TNF-α and IL-6), and thereby alleviate pulmonary edema in ALI models via down-regulation of WISP1. A previous research has already illustrated that IL-18 as well as caspase-1 plays critical roles in the development of lung injury like ARDS [29]. In addition, another study demonstrated that LPS-induced ALI rats express significantly up-regulated levels of pro-inflammatory cytokines TNF-α, IL-6, and IL-1β during lung injury [30]. Also, a number of miRNAs have been explained to be regulated dynamically in LPS induced ALI mice, among which miR-16 was significantly downregulated, and over-expression of miR-16 led to a reduction in IL-6 and TNF-α expression [31]. The promoting effects of WISP1 on the release of TNF-α in macrophages have also been documented in sepsis-induced lung injury, suggesting it functions as a contributor of inflammation [32]. Largely in agreement with our findings, over-expressed miR-145-5p has been revealed to diminish the high levels of TNF-α in BV-2 cells induced by over-expressed SNHG14 [22]. These results provide evidence supporting the regulatory mechanism of the lncRNA SNHG14/miR-34c-3p/WISP1 axis in ALI.

## Conclusion

**Fig. 6:**
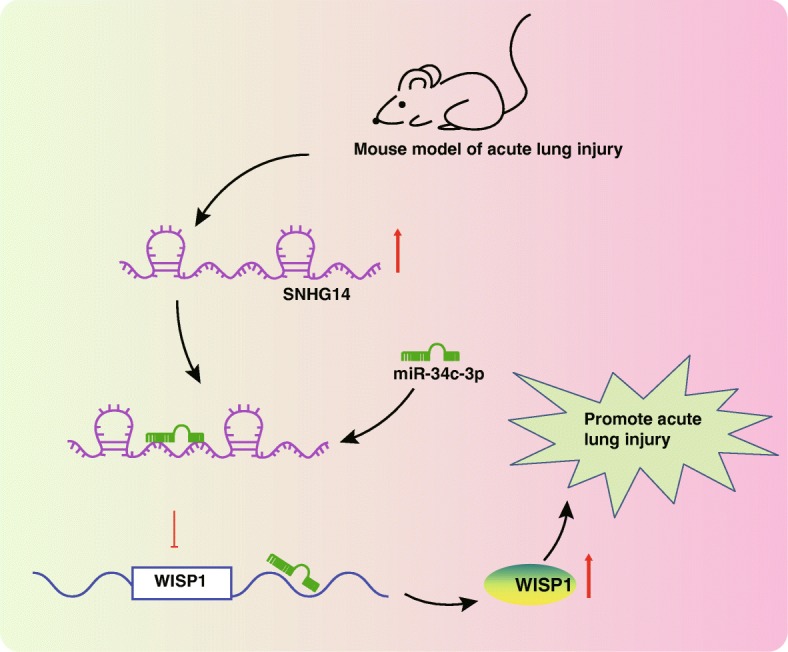
The schematic diagram showing the mechanisms responsible for the promotive role of lncRNA SNHG14 in ALI. LncRNA SNHG14 binds to miR-34c-3p to up-regulate WISP1. SNHG14 aggravates LPS-induced ALI via impairing miR-34c-3p-mediated WISP1 down-regulation

Returning to the initial hypothesis posed at the beginning of this study, it would be reasonable to state that lncRNA SNHG14 knockdown confers protection against ALI via miR-34c-3p-mediated down-regulation of WISP1. After lncRNA SNHG14 is down-regulated, the binding of miR-34c-3p to WISP1 is increased, leading to depletion of WISP1, thus alleviating ALI severity (Fig. 6). Therefore, lncRNA SNHG14 may serve as a novel therapeutic approach to ALI. However, genetic mismatch effects of alveolar macrophage cell line derived from BALB/c mice (MH-S cells) and ICR mice might limit the generalization of our findings. Nevertheless, the current study provides innovative perspectives to advance the hepatoprotective therapy. Further efforts are warranted to validate the generalization for effective clinical applications. Moreover, the interaction between extra-cellular terminally differentiated cells on endothelium has not been studied yet due to current limited conditions. This would be another interesting topic for future researches.

## Data Availability

The datasets generated/analysed during the current study are available.

## Abbreviations

ALI: Acute lung injury
ARDS: Acute respiratory distress syndrome
LPS: Lipopolysaccharides
NSCLC: Non-small cell lung cancer
WISP1: Wnt1-inducible signaling pathway protein-1
